# Preparation and Support of Patients through the Transplant Process: Understanding the Recipients' Perspectives

**DOI:** 10.1155/2012/547312

**Published:** 2012-10-17

**Authors:** Oliver Mauthner, Enza De Luca, Jennifer Poole, Mena Gewarges, Susan E. Abbey, Margrit Shildrick, Heather Ross

**Affiliations:** ^1^Cardiac Transplant Program, University Health Network, 585 University Avenue, NCSB 11-G31, Toronto, ON, M5G 2N2, Canada; ^2^School of Social Work, Faculty of Community Services, Ryerson University, 350 Victoria Street, EPH-220, Toronto, ON, M5B 2K3, Canada; ^3^Department of Psychiatry, University Health Network, 585 University Avenue, NCSB 11C-1114, Toronto, ON, M5G 2N2, Canada; ^4^Gender and Knowledge Production in the Medical, Technical and Natural Sciences, Tema Genus, Linköping University, 58183 Linköping, Sweden; ^5^Divisions of Cardiology and Transplant, University Health Network, 585 University Avenue, NCSB 11-1203, Toronto, ON, M5G 2N2, Canada

## Abstract

Preparation for heart transplant commonly includes booklets, instructional videos, personalized teaching sessions, and mentorship. This paper explores heart transplant recipients' thoughts on their preparation and support through the transplant process. Twenty-five interviews were audio-/videotaped capturing voice and body language and transcribed verbatim. Coding addressed language, bodily gesture, volume, and tone in keeping with our visual methodology. Recipients reported that only someone who had a transplant truly understands the experience. As participants face illness and life-altering experiences, maintaining a positive attitude and hope is essential to coping well. Healthcare professionals provide ongoing care and reassurance about recipients' medical status. Mentors, family members, and close friends play vital roles in supporting recipients. Participants reported that only heart transplant recipients understood the experience, the hope, and ultimately the suffering associated with living with another persons' heart. Attention needs to be focused not solely on the use of teaching modalities, but also on the development of innovative support networks. This will promote patient and caregiver engagement in self-management. Enhancing clinicians' knowledge of the existential aspects of transplantation will provide them with a nuanced understanding of the patients' experience, which will ultimately enhance their ability to better prepare and support patients and their caregivers.

## 1. Introduction

With heart transplantation comes well-documented medical and psychosocial challenges [1, 2]. Healthcare professionals are dedicated to educating and supporting transplant recipients and their families throughout this potentially difficult trajectory in order to promote engagement in self-management. Recipients are commonly prepared and supported during their transplant through a variety of modalities which include booklets, instructional videos, personalized teaching sessions, and peer mentorship. To better understand their needs, we asked patients participating in this research study to reflect on their experience of being a transplant recipient. This paper focuses on participants' experiences and their perspectives on how to prepare and support recipients through the transplant process, which is one component of a larger study [3]. Our interdisciplinary team employed a visual methodology which allowed us to interpret body gesture and spoken words with the understanding that images and words form a set of different representations highlighting emotions and experiences [4]. Such methodology is useful as it provides an innovative framework to engage with the complexity of transplantation [5].

## 2. Background

Patient and graft survival, transplant outcomes, and side effects [1], as well as the ethics of organ transplantation [6–10], have been extensively studied and will not be addressed in detail here. The average life expectancy after cardiac transplantation is 10 years with a conditional life expectancy of 13 years based on survival to year one [1]. A large number of qualitative studies show improvement in quality of life (QoL), but poor return to work (approximately 45%), as well as high general anxiety and distress among heart transplant recipients [1–3, 11, 12]. Dew and DiMartini's [13] review incorporating nearly 150 studies confirms that “depressive and anxiety-related disorders and associated distress are common post-transplant” and that such symptoms are not confined to the initial stages of recovery, but might appear or be exacerbated at any time” [page S51]. One longitudinal QoL study followed 156 patients over a four-year period post-transplant using the depression and anxiety subscales of the Symptom Checklist 90 [2]. Twenty-one percent experienced “high, clinically significant distress at all times” [2, page 1215]. Another 12% showed “high distress over several years with low distress only at final assessment,” whereas the remaining participants experienced low or fluctuating levels of distress for the duration of the study [2, page 1215]. Approximately one-third of heart recipients, therefore, were found to experience substantial, sustained distress. A multisite study investigating QoL in 555 individuals at 5–10 years post-heart-transplant found that depression accounted for the variance both in overall QoL and in health/functioning QoL [14].

In the transplantation literature, “distress” refers to psychiatric diagnoses such as depression, anxiety, and psychosis [15–22]. Psychoanalytic studies of recipients' adjustment to an organ graft have identified common responses and coping mechanisms, such as overpowering feelings of gratitude to the donor family, guilt over the donor's death, denial, and mourning of their own lost organ [23–32]. Research conducted at a major transplant center in Canada [3] with adult transplant recipients using qualitative research methods demonstrated that 88% of heart transplant patients experience pervasive post-transplant distress. Spaderna et al. [33] suggested that “social isolation, especially when combined with depression scores in the clinical range, may be important for the prognosis of heart transplant candidates” [page 252]. The authors reported that patients who died or had worsening clinical outcomes had small social networks [33]. A qualitative investigation by Kaba et al. [34] explored coping strategies of individuals following heart transplantation. Recipients adopted various forms of coping strategies, but frequently were searching for better ways to cope. Kaba et al. [34] highlighted the need for additional information for patients that outlines potential concerns post-transplant and strategies to address them.

Challenges are not limited to post-transplant experiences. Kop [35] highlighted that while waiting for a heart transplant, candidates experience a high level of psychological distress related to the potential unavailability of a donor heart and the life-threatening nature of heart failure. The value of psychological interventions in supporting candidates in maintaining positive coping mechanisms and a healthy lifestyle was identified by Kop [35], Spaderna et al. [33], and Zipfel et al. [36]. It is important to involve family and/or significant others in candidates' daily care, because they are at risk for social isolation and their emotional well-being impacts their disease trajectory following a heart transplant [35].

In a study conducted by Haugh and Sayler [37], the team reported that maintaining respect and dignity, being sensitive to family, sharing information, facilitating coping, and doing the “extra little things,” were helpful in supporting candidates who experience uncertainty while waiting for a donor heart. An investigation by Yorke and Cameron-Traub [38] provides an in-depth description of patients' perceptions of care from nursing staff while on a heart and lung transplant waiting list. The author of this investigation highlights that nursing staff are central to the care of patients waiting for a heart transplant, because they provide information, maintain regular contact, provide familiarity, and have a positive attitude and compassion [38, page 82].

Prior to discussing the study's methods, we outline the preparation potential transplant recipients currently undergo. At the Canadian academic teaching hospital where the research was conducted, patients being listed for heart transplant are prepared for the process in a variety of ways. The first conversation about heart transplantation occurs with a cardiologist. A nurse practitioner meets with them to provide information about what to expect while waiting for, and life after, transplant. Patients are also provided with a large educational manual and a videotape that explains the biomedical aspects of the transplant in lay terms. They then meet with a social worker to discuss medication regimens, their social support network, employment, insurance, and available financial supports. Each potential recipient is offered a transplant mentor to provide support outside the program although not all choose to have one [39]. While there is a formal process to become a mentor, how the relationship unfolds and what it provides each participant are left entirely up to the patient and the mentor. There is no predetermined structure to the mentor/mentee relationship and having a mentor is not a requirement for recipient listing. This preparation program has not been formally evaluated to determine which of these patient education modalities are most effective for individuals awaiting heart transplants.

## 3. Methods

An interdisciplinary team, composed of two advanced practice nurses, a cardiologist, a social worker, a sociologist, a psychiatrist, and a philosopher, engaged in a qualitative research study. The study is descriptive and exploratory. As per visual methodology, interviews of heart transplant recipients were digitally audio and video recorded [3]. Our visual methodology is oriented to the work of existential phenomenologist Merleau-Ponty [40, 41]. Like Kvigne et al. [42], we begin from the basis that existential phenomenology is not a research method. Rather, it is an orientation and sensitivity that both text and body language are central to understanding and analysis [42]. This means that we are not situating our study in the phenomenological tradition. Visual methodology is a distinct body of research in the field of sociology that is concerned with ways in which bodily conduct and talk are both important characteristics in social interaction [4]. The assumption is that images and words form a set of different representations highlighting emotions and experiences [4]. In our research, this implies incorporating an analytic approach that makes meaningful links between various experiences, visual data, and other objects. These two forms of media represent different types of knowledge that might be understood in relation to one another [4].

Members of our unique interdisciplinary research team have extensive formal training in conducting qualitative research. We bring together professors, scholars, and senior scientists in both the field of medicine and social sciences who all bring unique expertise and contributions to this research project. Informed by the work of Christian Heath and Sarah Pink who are both well known for their use of visual methodology, our team designed our unique visual methodology. We conducted pilot interviews to test our methodology, specifically our analysis process. We have published a number of papers that demonstrate how we have used video in concert with in-depth interviewing and field notes [3, 43, 44].

The study was approved by the Research Ethics Board (no. 07-0822-BE) and took place in a heart transplant program of a large metropolitan hospital in the southern portion of an eastern province in Canada. All individuals who met inclusion criteria were consecutively approached by a transplant nurse not affiliated with the study. This was done so that potential study participants had the opportunity to decline to take part in the study and not feel obligated to the investigators. When a potential participant showed interest in the study, a research associate discussed the study in detail and obtained informed consent. Thirty-six patients were approached, 6 declined to participate, and 3 did not follow through after they signed the consent. Sample size was in keeping with similar qualitative research studies and recruitment was stopped when we reached theoretical saturation.

Each patient provided written, informed consent prior to participation. The study included 27 post heart transplant patients, 2 videos were technically compromised (audio recording failure) which yielded 25 analyzable videos (70% men, mean age 53 yrs (±13.8), range 18–72; mean time since transplant 4.1 yrs (±2.4); 20 White, 2 Black, 5 South Asian). Participants were at least 18 years old, 1–10 years post-transplant, English speaking, and medically stable. Regardless of the time elapsed since transplantation, anecdotal and clinical involvement with transplant recipients tells us that they are able to communicate their experiences with immediacy and clarity. We accept as true that memory is a process, and as such, all participant responses reflect their embodied experiences and enriched and informed our findings accordingly.

Individuals were given a choice of where their interview would take place. All were conducted in nonclinical settings, mostly in their homes (*n* = 19). A few chose hospital based conference or “sitting” rooms that were distinct from the clinic area (*n* = 8). Each interview was audio and videotaped to capture voice and body language concurrently. All participants received instructions on how to switch off the recording equipment at any time. The camera was purposely visible and static to film the embodied interaction of the interviewer and participant, a practice consistent with standard contemporary visual methods [45].

The use of a video camera in research has been widely debated [46–48]. Some argued that having a camera present has negligible or no impact on participants [49–51]. Researchers have also suggested that video methods are valid only if used secretly or in triangulation with other methods which reduce “contamination” of the data such as respondent validation [6, 8, 19–22]. Yet given our phenomenological orientation, we argue that these positions are not only ontologically and epistemologically incompatible with our process, but also “run the risk of blinding themselves to the advantages of videoing as a method” [25, page 1154]. As visual methodologists, we espouse that we cannot observe the world without being present in it; hence, we believe that the use of camera was not neutral in the interview, but rather was a fundamental part of knowledge production [3]. During the interviews, the completely exposed camera became inconspicuous for both the interviewer and the participant. No participants chose to interrupt or stop the video recording at any time.

The interviews were conducted individually by one of two advanced practice nurses with extensive training in qualitative research and no involvement in the study participants' clinical care, on average four and a half weeks after recruitment. Approximately 30 minutes was spent prior to each interview answering questions about the research, establishing rapport, and building trust with the participants [52]. Participants were informed that they could refuse to answer any question, stop the interview at any point, or request erasure of anything they said. Following standard procedures in semistructured qualitative studies, the interviewer asked participants open-ended questions and provided opportunities for them to raise their own concerns. For example, participants were asked both “How would you prepare someone for transplant?” and “How would you support someone after transplant?” Techniques such as nodding, allowing silences, and using phrases such as “would you feel comfortable telling me more about that?” were utilized when necessary [53]. Interviews took 30–90 minutes. Following every interview, the researcher compiled detailed field notes of observations. Coding conventions established by Poland and Pederson [54, 55], such as noting laughter, silences, and pauses in addition to dialogue, were used to professionally transcribe audio files verbatim. Digital video files, transcripts and field notes, were imported into the NVivo8 qualitative research software program.

All interviews were viewed in their entirety by the research team. The team met as a group together in the same room throughout the analysis process. The video and transcripts were analyzed simultaneously and in an iterative process. Coding was informed by the work of visual methodologists Pink [4, 56] and Heath [45, 57], using NVivo8 to organize the data. The first phase of data analysis started with a transcript and videotape review in which they were simultaneously time logged, and “key moments” were noted [4]. Key moments included particular statements in transcripts and videotaped embodied responses such as “expressive gestures” [57, 58] that located “areas of difficulty” on/in the body (e.g., hands on heart; pointing to the heart) [57, 58], “expressive artifacts” (e.g., open necked shirts showing surgical scars), and “by the way syndrome”, that is, the gestures and comments close to the end of interviews that (re)asserted the “significance or seriousness of a particular symptom/feeling” [57, 58]. Finally, “incongruities” between participants' words and gestures were noted [57, 58]. These include but are not limited to “upgrades,” which represent speaking positively even when body comportment indicated distress (i.e., inability to maintain eye contact; crying; intense fidgeting), or “downgrades” when comportment appeared nondistressed (i.e., calm tone of voice; relaxed posture) even though words revealed the opposite [57, 58].

In the next phase of data analysis, all audio visual data were collectively re-reviewed. Broad themes were developed by the team from transcript quotes and audio visual footage and where tagged to key moments. These themes were defined, discussed, debated, and agreed on by the team before being collapsed into analytic categories. The team then collapsed these analytic categories into a final set of themes which addressed the research question [3]. To meet the highest standards of methodological rigor, an audit trail was maintained (specific record of methodological and data coding decisions). The flexible storage, cross-indexing, and quick retrieval features of NVivo8 made it easy to search for negative instances and universal findings [59]. Attention was paid to data inconsistent with overall findings, and coding was discussed with all members of the team. Issues not resolved through consensus led to discussion and further analysis.

## 4. Results

In reporting data in visual methods research, we are unable to use video footage to show our findings; hence in this paper we rely solely on words to discuss results. We have therefore adopted a very descriptive writing style to compensate for what cannot be seen. The aforementioned analysis processes led to the identification of several themes. The most common theme was recipients' sense of not being fully understood. Participants talked about that only someone who has gone through this process truly understands the complexity of their experiences. Other themes included the need to maintain hope, where a positive attitude was seen as essential in order to move forward. Participants reported that ongoing close connections with healthcare professionals (HCPs) provided reassurance about their ongoing medical care. The presence of family and friends was also seen as essential, as was their help in managing day-to-day activities. Mentorship programs provided a different and unique form of support between individuals who may share an understanding or experience. The mentor-mentee relationship is often developed between an individual who is awaiting transplant and a transplant recipient.

### 4.1. Not Understood

Fourteen patients (56%) commented that only heart transplant recipients truly understood the experiences they were going through. Hence, transplant recipients were most able to prepare them for transplantation and support them afterwards. Participants expressed that living through heart transplantation cannot be compared to any other medical condition. They reported it was a very emotional, distressing, and physically demanding. To this end, all recipients felt their transplant experience was unique.

A thirty-year-old woman, who was interviewed in a room close to the heart transplant clinic area, slouched down in her chair while she said “… I do not like to tell people unless they've been through something similar, because the truth is that you cannot really understand unless you've been through the same thing… (PTx13).” She seemed to have reconciled herself to the idea that other people just would not understand, and she had told few people about her ordeal. A fifty-six-year-old man, who was interviewed in his home, sat crossed legged on a couch with closed posture. In a very monotone voice he spoke about what he would say to a potential recipient as follows: “… look, if you are really in a bad way, here is my phone number, here is my email…. I know exactly what you are going through, I know how bad it gets, you cannot surprise me, just phone… (PTx12).” He communicated this in a voice devoid of inflection and as if he had rehearsed this message. Another man in his early thirties, sitting at his kitchen table, used expressive gesture and voice said “… I think education is important but sometimes you also have to live through it to understand it (PTx21).” His message had a sense of urgency, emphasizing the difficulties associated with not being understood. A man in his late fifties sitting at his kitchen table stated “You know, you guys [transplant team] think you know what we went through, but you do not (PTx10).” The difficulty for him in communicating this was apparent in his low and flat tone of voice as he nervously twirled his fingers and avoided eye contact.

### 4.2. Maintaining Hope

Maintaining their positive attitude after transplant was reportedly very important for 13 (52%) of the recipients. Their statements included endorsements of not being told about potential transplant-related complications or personal struggles and receiving only encouraging words. A fifty-four-year-old woman, who was interviewed at a large table in a dimly lit kitchen, said with a very worried look on her face “Well, you have to encourage this person… never tell them… Oh, my God, you know, tomorrow you are going to be worse, never say that to the person who went through a nightmare… never discourage people (PTx15).” As the inflection in her voice rose, in an effort to make her point clear, she wrapped her arms around her body, as if comforting herself while sharing her difficult story. Nervously giggling, a man, who had spoken previously in his interview of the emotional difficulty of going through a heart transplant, said that if he were to speak to another patient, he would say “Do not worry about it, everything is going to be fine… I would tell him that he is going to feel good after. He is going to feel better than before… (PTx17).” As he completed his statement, he leaned forward with open arms to enforce that although his experience was difficult, he would only share positive outcomes with other potential recipient.

### 4.3. Ties to the Team

Thirteen (52%) recipients commented that HCPs had provided the needed reassurance about their ongoing medical status and continuity between the transplant clinic and their home. A fifty-six-year-old man who was seven years post-transplant stated “my support system, I would have to say, are the transplant nurse coordinators because if ever there is a problem I could just call them, and they do phone back… I just feel very protected and well taken care of… whenever I had questions, all I needed to do was call, and they were answered (PTx12).” At this point in his interview, the participant relaxed his posture and seemed to speak more freely about his experience. Another male participant stated that “… they [HCP] are there if you need them, but like I say, first couple of years you really depend on them, you feel so much better going there [hospital]…. The transplant team is really extraordinary. I do not know how they do it… (PTx10).” He made this statement with a clear assertive voice and repeated it more than once. Another young man reported “My support system, I would have to say it's [Advanced Practice Nurses' names], because if ever there is a problem, you know, I can just call them… I think in the whole that I just feel very, uhm, protected and well taken care off from there [hospital], so whenever I have problems… questions, all I needed to do was call… (PTx22).” He represented this in a very matter of fact fashion, as if the answer was obvious.

### 4.4. Family and Friends

Family members and close friends were reported by 5 (20%) recipients to have been an important part of their lives throughout the transplant experience. Sitting very composed with crossed legs, a male interviewee flatly said “[wife's name] was there all the time, right with me, and there were a couple of really good friends who just made sure I was OK… (PTx12).” He spoke as if his answers had been scripted, using a very monotonous tone of voice and a very controlled body posture. A physically fit looking man who sat at a table stated “I think they [recipients] need to have somebody around because the days are long… they need to have somebody around that can, you know, they can gab with and hang out with, so you do not have all that time to think (PTx14).” A woman, sitting in a large, well-appointed living room stated “There are lots of people in my life who have been really great trying to help me out. But mostly my husband, he has been a saint, a saint…. I honestly do not know how somebody could get through it alone. If I had not had my husband, I would not have made it. You just… you cannot. It is so hard to do alone (PTx24).” She delivered her message with a lot of emphasis and certainty; there was conviction in her voice. She continued by saying “… you just have to be there for them [transplant recipients]. I would imagine that driving people around would be all part of it, and being with them. Some of it is you need them there to do stuff for you, another is to… commiserate with you (PTx24).”

### 4.5. Mentorship

The transplant program offers every patient the opportunity to connect with a mentor, who is a transplant recipient. When participants were asked how to best support transplant recipients, 10 (40%) spontaneously spoke of this mentorship program. Six of those 10 recipients reported having elected to be mentored and described it as a supportive experience. The 4 participants, who had declined having a mentor, retrospectively thought being a mentee would have been beneficial. A frail looking older married woman, sitting at her kitchen table, said how helpful it had been “… because you always wonder what it is going to be like after the transplant, and seeing someone by your bedside that has already had a heart transplant… just saying everything is going to be fine… (PTx9).” As she said the words “everything is going to be fine,” her body changed from being slouched forward with arms crossed and she became more engaged by using expressive gestures and increasing the tone and inflection of her voice. A retired man, who reported during his interview that the support of mentors was vital in his recovery, nervously chuckled as he said “I did hear a lot of people saying that they did not have much support as far as mentors… it's a big thing [transplant], it's too bad…. I think the mentor thing is really good (PTx10).” Another man appeared content and comfortable sitting in his living room stated, “They set you up with a buddy system…. We met up and had a chat… its nice to see somebody that's had a transplant… I do not think anybody said too much negative, the only thing is I think they all, after a few years, forget the sort of bit of trauma you go through… (PTx11).” He leaned comfortably back in his chair, crossing his hands in his lap.

## 5. Discussion

Many participants speak about the difficulty of living with a transplanted heart. They stressed that most “others” do not understand what recipients go through and that only someone who has a heart transplant truly understands the experience. Similar findings have been reported by Sadala and Stolf [60]. Our effort to uncover what “we do not understand” led us to further explore why transplant recipients think that only someone who has lived through the transplant experience understands them. Conventional research methods have not been comprehensive or sensitive enough to understand the multifaceted aspect of patient experience because the focus has been on the written or spoken word. Such methods ask participants to speak or enumerate their experience of heart transplantation but fail to illuminate what cannot be spoken. Hence, the unspoken ultimately remains hidden from the researcher. As described by visual methodologist Heath [58], suffering can be seen in the body through expressive gestures and tone of voice. The use of visual methods enables researchers to interpret the expressive body, making visible what would otherwise remain hidden. Embodied suffering is revealed through visual methods. Its use in the study reported here enabled us to innovatively engage with transplant recipients' unique experiences and gain new insights into their ongoing suffering.

To this end, the interviews captured the very body showing everything the mind suffers: crying, moaning, lacking affect, dropping their tone of voice, speaking monotonously, stooped shoulders, head dropped forward, avoiding eye contact and legs crossed. In this paper, the notion of suffering captures participants' affective experience of sadness or unpleasantness. Heath [58, page 603] reports that “through gesture and bodily conduct, patients transpose inner suffering, their personal subjective experience of their complaint, to the body's surface and particular parts and areas of their physic”. As described in our findings, the interviewees' bodily comportment characterizes the difficulty of receiving a heart transplant allowing the research team to witness their suffering. In order to better support our recipients, we turned to the work of Frank [61, page 355] to gain further insight about suffering.

**Suffering is the unspeakable, as opposed to what can be spoken; it is what remains concealed, impossible to reveal; it remains in darkness, eluding illumination; and it is dread, beyond what is tangible even if hurtful. Suffering is loss, present or anticipated, and loss is another instance of no thing, and absence…. Suffering resists definition because it is the reality of what is not.**

If suffering is “unspeakable,” it explicates why more than half of the study participants felt only someone who has walked, lived, and suffered in their shoes could understand their plight [61]. It also demonstrates how the experience of post-transplant suffering might remain hidden from professionals, defying language and more standard research methods. Taking this analysis further, it is possible to look at a wound as a metaphor for suffering [61]. A physician might look at the wound in diagnostic/clinical contexts (i.e., healing, infection), whereas for the patient, the wound is something experienced, felt, seen, and smelled. Health care practitioners look at heart transplantation through a clinical/diagnostic lens. Recipients are assessed and measured: weight, blood pressure, and body temperature are recorded, blood work is reviewed, and biopsies are performed and examined—all to ensure that the transplanted organ remains healthy and that the individual's body is not rejecting the heart. Healthcare practitioners also value recipients' psychological well-being and the importance of quality of life. Yet recipients continue to report anecdotally, and in the study reported here, that most clinicians do not understand them, and yet they play an essential role in providing support related to the medical management. There seems to be a divide between bodies that receive transplants and transplant recipients' lived experience of these bodies. Given that the recipients' lived experiences are private, personal, and often indescribable, they are not accessible to HCPs. This disconnect cannot be overcome solely by asking recipients what their experience of living with a transplanted heart is like. As highlighted by Frank [61], when patients are asked to talk about difficult experiences such as their transplantation, they are unable to fully articulate their experience and tend to express it through particular complaints and concerns. What remains concealed is their suffering. Frank [61] best describes this when he says “suffering is expressed in myth as the wound that does not kill but cannot be healed” [page 355]. It follows that until patients' suffering is understood, this existential wound cannot heal. Each recipients' suffering is rooted in their embodied experience, and as long as HCPs do not engage with their embodied lived distress, a disconnect will continue. Our intent was to find ways to enhance clinicians' knowledge of and ability to provide appropriate support. When asked how to best do this, participants reported that providing encouragement and not focusing on potential negative disease sequelae were very important to maintain hope.

Hope was described as an essential component in dealing well with illness and life-altering experiences. Wiles et al. [62] performed a narrative literature review about hope, expectations, and recovery from illness. They highlighted that it is essential to understand the function of hope when dealing with illness and the recovery process as it “provides a coping mechanism in the face of what people may experience as the otherwise intolerable impacts of a health crisis, and maybe a common adaptive response… [62, page 569].” They describe hope as having two components: hope as an “expectation” and hope as a “want.” Within the context of the research presented here, hope as an expectation in heart transplant recipients includes hoping that specific symptoms or events will not occur during one's disease trajectory. Alternately, hope as a want is less likely to be realized. A heart transplant recipient might hope to meet the donor family or return to his/her old “self.” To this end, our research team acknowledges that HCPs should be aware of this nuanced understanding of hope to better support the patient, because it is conceivable that false hope could adversely affect recovery.

Participants identified three major sources of support: their transplant team, family and friends, and transplant mentors. These findings are in keeping with reports from Sadala and Stolf [60]. When conceptualizing what participants discussed about support, we draw on the work of King et al. [63] who studied social support processes of individuals with chronic conditions. King et al. [63] described distinct categories of psychological support, including “instrumental support” (allowing participants to achieve self-efficacy through direction and planning of approaches) and “emotional support” (being valued and accepted provides participants the sense of “being believed in”) [page 915]. These themes are consistent with participants' accounts in our study and will guide the following discussion.

Participants reported that health care professionals provided “instrumental support” to transplant recipients through ongoing assessment of health status and guidance in their self-management of care. In doing such the HCPs also provided emotional support to individuals. Participants were able to review their concerns with transplant professionals during clinic visits as well as through a sophisticated patient management telephone system, providing them not only with access to resources, but also with ongoing reinforcement to be able to manage their own care. This type of support provided transplant recipients with strategies to ultimately enhance their competence taking on the onerous tasks of self-management. Such tasks include managing their medications, medical challenges, and adapting to their altered life with a transplanted organ.

For some transplant recipients, the mentor/mentee relationship has continued throughout the transplant trajectory. The ability for transplant recipients to connect with individuals, who have experienced a similar process, provides an opportunity for the provision of “emotional support.” The mentor/mentee relationship offers a unique connection between transplant recipients that creates a sense of belonging. King et al. [63] describe that when “emotional support” is provided, people feel accepted and trusted, promoting a sense of “being believed in.” Hence, mentors who have been through a similar experience, allow transplant recipients to feel understood. Mentor/mentee relationships afford an additional benefit, in that providing and receiving support can be mutually beneficial [64]. In supporting another recipient, the mentor gains a sense of meaning and satisfaction, which in turn might improve adaptation to their own illness.

The presence and availability of family and friends provided great comfort to some transplant recipients. Assistance with day to day responsibilities and having others around even if they are “just being there” are both sources of comfort and support [65]. Study participants talked about receiving support with household chores and help with tracking medications and medical appointments. Having a family member or a friend present during clinic visits and invasive procedures similarly provided support to transplant recipients. It is important for HCPs to recognize the significance and complexity of the role that family and friends might play during the heart transplant recipient's life trajectory.

## 6. Study Limitations

Participants, although ethnically and socioeconomically diverse, were all recruited from a single academic health care setting. They were recruited consecutively as they attended routine follow-up clinics to minimize bias. Our study population represents sex, ethnic, and social demographic groups that are comparable to our larger urban transplant program population. Both interviewers are researchers with this study, actively participating in all aspects of the research process, and are the main authors of this manuscript. Although all study participants were asked the same questions, in keeping with conventions of semistructured interviews, the discourse of the interview was not directed by the interviewer, allowing the conversation to evolve spontaneously. Consequently, not all participants spoke of the same topics, limiting the ability to draw conclusions about participants who did not report on certain themes.

## 7. Conclusion

Participants felt that few people understood what they were going through and that only someone who has had a heart transplant truly understood the experience, the suffering, the hope, and a second chance at life. If Frank is correct, because of the unspeakable nature of suffering, key issues of the transplant experience remain hidden from the HCP and most researchers. Visual methods provide a portal to more comprehensively engage with the notion of suffering. They might allow researchers and ultimately HCPs to understand and hence acknowledge heart transplant recipients concerns, thereby giving their experiences legitimacy. Also, knowledge of the existential aspects of organ transplantation will provide HCPs with a nuanced understanding of the patients' transplant experience. This will allow them to better prepare and support patients through the transplant process, and ultimately promote patient and caregiver self-management.

In our research, we found that despite others inability to truly understand the transplant experience, ongoing relationships with family, friends, and healthcare providers were none-the-less fundamental in the provision of continuous support. In an effort to build stronger support networks, we also need to look at innovative ways to educate individuals who will be providing support for transplant recipients. Written teaching materials and instructional videos should not be the exclusive recipient preparation modalities because interpersonal relationships and interactions, including the mentorship program, were considered more helpful and supportive. Strong efficacious mentorship programs need to be developed and evaluated, recipient support networks need to be encouraged, clinicians' knowledge of the existential aspects of organ transplantation need to be enhanced.
